# Exact Multisoliton Solutions of General Nonlinear Schrödinger Equation with Derivative

**DOI:** 10.1155/2014/593983

**Published:** 2014-06-12

**Authors:** Qi Li, Qiu-yuan Duan, Jian-bing Zhang

**Affiliations:** ^1^Department of Mathematics, East China Institute of Technology, Nanchang 330013, China; ^2^Department of Mathematics, Fuzhou Vocational College of Technology, Fuzhou 344000, China; ^3^School of Mathematics and Statitics, Jiangsu Normal University, Xuzhou, Jiangsu 221116, China

## Abstract

Multisoliton solutions are derived for a general nonlinear Schrödinger equation with derivative by using Hirota's approach. The dynamics of one-soliton solution and two-soliton interactions are also illustrated. The considered equation can reduce to nonlinear Schrödinger equation with derivative as well as the solutions.

Some nonlinear partial differential equations are integrable models with interesting physical applications. Much work has been focused on those equations such as the celebrating KdV, modified KdV, nonlinear Schrödinger equations, and Toda lattice. Inverse scattering transform (IST), Darboux transformation, Hirota's approach, tanh-function method, and algebraic-geometry method [1–25] have been used to investigate the exact solutions and integrability of those equations. Among these methods, Hirota's approach is usually used to find *N*-soliton solutions for soliton equation. The key is transforming the soliton equations to the bilinear ones by introducing bilinear derivative and appropriate variable transformation [4]. The Hirota approach has been generalized to much more general bilinear equations recently [5]. The invariant subspace method is refined to present more unity and more diversity of exact solutions by taking subspaces of solutions to linear ordinary differential equations as invariant subspaces that evolution equations admit [6].

Associating with Kaup-Newell (KN shortly) spectral problem, there exist three types of derivative nonlinear Schrödinger equations [7, 8]. Gauge transformations have been found among them [9, 10]. The first well-known derivative nonlinear Schrödinger equation (DNLSE) is

(1) $$\begin{array}{c} \;iu_{t}+u_{xx}+i{\left(u^{2}u^{*}\right)}_{x}=0, \end{array}$$

where u* denotes the complex conjugate of *u*. This equation models Alfven waves and magnetohydrodynamic waves in plasmas and also model subpicosecond or femtosecond pulses in single-mode optical fibers in nonlinear optics [11, 12]. The equation is investigated in some literature (see, e.g., [13–15]). Its explicit form of the *N*-soliton solutions is also obtained by some algebraic technique [16]. Through the *n*-fold Darboux transformation the rogue wave solutions are constructed explicitly by seed solutions recently [17]. The standard NLS equation has a tri-Hamiltonian structure [18] and DNLSE equations has some sl(2) generalizations [19] and an so(3) generalization [20].

In the paper, we consider general nonlinear Schrödinger equation with derivative (GDNLSE) as follows:

(2a) $$\begin{array}{c} q_{t}=q_{xx}-{i\left(q^{2}r\right)}_{x}, \end{array}$$

(2b) $$\begin{array}{c} r_{t}=-r_{xx}-{i\left(qr^{2}\right)}_{x}. \end{array}$$

We show that through a variable transformation the bilinear equations for (2a) and (2b) can be derived for constructing its *N*-soliton solutions. We also describe that the multisoliton solutions of (1) can be derived by reduction.

Firstly, we deduce the Lax pair of GDNLSE (2a) and (2b), which usually assures the complete integrability of a nonlinear equation. From the Kaup-Newell spectral problem

(3a) $$\begin{array}{c} {\left(\begin{array}{c} \phi_{1}\\ \phi_{2} \end{array}\right)}_{x}=M\left(\begin{array}{c} \phi_{1}\\ \phi_{2} \end{array}\right),\;M=\left(\begin{array}{cc} -i\eta^{2} & \eta q\\ \eta r & i\eta^{2} \end{array}\right), \end{array}$$

time evolution

(3b) $$\begin{array}{c} {\left(\begin{array}{c} \phi_{1}\\ \phi_{2} \end{array}\right)}_{t}=N\left(\begin{array}{c} \phi_{1}\\ \phi_{2} \end{array}\right),\;N=\left(\begin{array}{cc} A & B\\ C & -A \end{array}\right), \end{array}$$

and the related zero curvature equation

(4) $$\begin{array}{c} M_{t}-N_{x}+\left[M,N\right]=0, \end{array}$$

one can derive the GDNLSE (2a) and (2b). Its corresponding Lax pair (3a) and (3b) is governed by

(5a) $$\begin{array}{c} A=-2\eta^{4}-\eta^{2}qr, \end{array}$$

(5b) $$\begin{array}{c} B=-2i\eta^{3}q+\eta \left(q_{x}-iq^{2}r\right), \end{array}$$

(5c) $$\begin{array}{c} C=-2i\eta^{3}r-\eta \left(r_{x}+iqr^{2}\right). \end{array}$$

Secondly, we give the bilinear form of GDNLSE and further its *N*-soliton solutions. By the variable transformation

(6) $$\begin{array}{c} q=\frac{gs}{f^{2}},\;\;r=\frac{hf}{s^{2}}, \end{array}$$

GDNLSE (2a) and (2b) can be transformed to the bilinear form

(7a) $$\begin{array}{c} \left(D_{t}-D_{x}^{2}\right)g\cdot f=0, \end{array}$$

(7b) $$\begin{array}{c} \left(D_{t}+D_{x}^{2}\right)h\cdot s=0, \end{array}$$

(7c) $$\begin{array}{c} \left(D_{t}-D_{x}^{2}\right)f\cdot s=0, \end{array}$$

(7d) $$\begin{array}{c} D_{x}f\cdot s=-\frac{i}{2}gh, \end{array}$$

where *g*,  *h*,  *f*, and *s* are complex functions and *D* is the well-known Hirota bilinear operator defined as

(8) DtmDxna·b=(∂t−∂t′)m(∂x−∂x′)na(t,x)b(t′,x′)|t′=t,x′=x.

To solve the system (7a), (7b), (7c), and (7d), we expand *f*,  *g*,  *h*, and *s* as

(9) $$\begin{array}{c} f=1+\overset{\infty }{\underset{j=1}{\sum }}f^{\left(2j\right)}\varepsilon^{2j},\;\;g=\overset{\infty }{\underset{j=1}{\sum }}g^{\left(2j-1\right)}\varepsilon^{2j-1},\\ h=\overset{\infty }{\underset{j=1}{\sum }}h^{\left(2j-1\right)}\varepsilon^{2j-1},\;\;s=1+\overset{\infty }{\underset{j=1}{\sum }}s^{\left(2j\right)}\varepsilon^{2j}. \end{array}$$

Substituting (9) into (7a), (7b), (7c), and (7d) yields

(10a) $$\begin{array}{c} g_{t}^{\left(1\right)}-g_{xx}^{\left(1\right)}=0, \end{array}$$

(10b) $$\begin{array}{c} g_{t}^{\left(3\right)}-g_{xx}^{\left(3\right)}=\left(-D_{t}+D_{x}^{2}\right)g^{\left(1\right)}\cdot f^{\left(2\right)}, \end{array}$$

(10c) $$\begin{array}{c} g_{t}^{\left(5\right)}-g_{xx}^{\left(5\right)}=\left(-D_{t}+D_{x}^{2}\right)\left(g^{\left(1\right)}\cdot f^{\left(4\right)}+g^{\left(3\right)}\cdot f^{\left(2\right)}\right), \end{array}$$

(11a) $$\begin{array}{c} h_{t}^{\left(1\right)}+h_{xx}^{\left(1\right)}=0, \end{array}$$

(11b) $$\begin{array}{c} h_{t}^{\left(3\right)}+h_{xx}^{\left(3\right)}=-\left(D_{t}+D_{x}^{2}\right)h^{\left(1\right)}\cdot s^{\left(2\right)}, \end{array}$$

(11c) $$\begin{array}{c} h_{t}^{\left(5\right)}+h_{xx}^{\left(5\right)}=-\left(D_{t}+D_{x}^{2}\right)\left(h^{\left(1\right)}\cdot s^{\left(4\right)}+h^{\left(3\right)}\cdot s^{\left(2\right)}\right), \end{array}$$

(12a) $$\begin{array}{c} f_{t}^{\left(2\right)}-f_{xx}^{\left(2\right)}-\left(s_{t}^{\left(2\right)}+s_{xx}^{\left(2\right)}\right)=0, \end{array}$$

(12b) $$\begin{array}{c} f_{t}^{\left(4\right)}-f_{xx}^{\left(4\right)}-\left(s_{t}^{\left(4\right)}+s_{xx}^{\left(4\right)}\right)=\left(-D_{t}+D_{x}^{2}\right)f^{\left(2\right)}\cdot s^{\left(2\right)}, \end{array}$$

(12c) ft(6)−fxx(6)−(st(6)+sxx(6)) =(−Dt+Dx2)(f(4)·s(2)+f(2)·s(4)),

(13a) $$\begin{array}{c} f_{x}^{\left(2\right)}-s_{x}^{\left(2\right)}=-\frac{i}{2}g^{\left(1\right)}h^{\left(1\right)}, \end{array}$$

(13b) $$\begin{array}{c} f_{x}^{\left(4\right)}-s_{x}^{\left(4\right)}=-D_{x}f^{\left(2\right)}\cdot s^{\left(2\right)}-\frac{i}{2}\left(g^{\left(1\right)}h^{\left(3\right)}+g^{\left(3\right)}h^{\left(1\right)}\right), \end{array}$$

(13c) fx(6)−sx(6)=−Dx(f(2)·s(4)+f(4)·s(2)) −i2(g(1)h(5)+g(3)h(3)+g(5)h(1)),

In order to get one-soliton of GDNLSE (2a) and (2b), we select *g* ^(1)^ and *h* ^(1)^ for (10a) and (11a) as follows:

(14a) $$\begin{array}{c} g^{\left(1\right)}=e^{\xi_{1}},\;\;\xi_{1}=\omega_{1}t-k_{1}x+\xi_{1}^{\left(0\right)},\;\;\omega_{1}=k_{1}^{2}, \end{array}$$

(14b) $$\begin{array}{c} h^{\left(1\right)}=e^{\eta_{1}},\;\;\eta_{1}=\sigma_{1}t+l_{1}x+\eta_{1}^{\left(0\right)},\;\;\sigma_{1}=-l_{1}^{2}, \end{array}$$

where *ξ*_1_^(0)^,  *η*_1_^(0)^ are all constants. Substituting (14a) and (14b) into (13a), one can obtain

(15) $$\begin{array}{c} f_{x}^{\left(2\right)}-s_{x}^{\left(2\right)}=-\frac{i}{2}e^{\xi_{1}+\eta_{1}}. \end{array}$$

Then combining (15) with (12a) yields

(16a) $$\begin{array}{c} f^{\left(2\right)}=\frac{ik_{1}}{2{\left(l_{1}-k_{1}\right)}^{2}}e^{\xi_{1}+\eta_{1}}, \end{array}$$

(16b) $$\begin{array}{c} s^{\left(2\right)}=\frac{il_{1}}{2{\left(l_{1}-k_{1}\right)}^{2}}e^{\xi_{1}+\eta_{1}}. \end{array}$$

Assuming that *g* ^(*i*)^ = *h* ^(*i*)^ = *f* ^(*j*)^ = *s* ^(*j*)^ = 0, (*i* = 3,5, 7,…, *j* = 4,6, 8,…), one can find that (10a), (10b) and (10c)–(13a), (13b), and (13c) are still hold. Thus, let *ϵ* = 1, substituting (9), (14a) and (14b) and (16a) and (16b) into (6), one can arrive at one soliton solution for GDNLSE (2a) and (2b):

(17) q1=eξ1(1+(l1/2)eξ1+η1+(π/2)i+θ13)(1+(k1/2)eξ1+η1+(π/2)i+θ13)2,r1=eη1(1+(k1/2)eξ1+η1+(π/2)i+θ13)(1+(l1/2)eξ1+η1+(π/2)i+θ13)2,

where *ξ*_1_, *η*_1_ are defined by (14a) and (14b), *k*_1_, *l*_1_ are all arbitrary constants, and *e* ^*θ*_13_^ = (1/(*l*_1_ − *k*_1_)^2^). We depict |*q*_1_| and |*r*_1_| in Figure 1. For convenience, we replace *t* by −*it* and *x* by −*x*.

To get two-soliton of GDNLSE (2a) and (2b), we select *g* ^(1)^ and *h* ^(1)^ for (10a) and (11a) as follows:

(18a) g(1)=eξ1+eξ2,  ξj=ωjt−kjx+ξj(0), ωj=kj2,(j=1,2),

(18b) $$\begin{array}{c} h^{\left(1\right)}=e^{\eta_{1}}+e^{\eta_{2}},\;\;\eta_{j}=\sigma_{j}t+l_{j}x+\eta_{j}^{\left(0\right)},\;\sigma_{j}=-l_{j}^{2}, \end{array}$$

where *ξ*_*j*_^(0)^,  *η*_*j*_^(0)^ are all constants. Substituting (18a) and (18b) into (13a), one can obtain

(19) $$\begin{array}{c} f_{x}^{\left(2\right)}-s_{x}^{\left(2\right)}=-\frac{i}{2}\left(e^{\xi_{1}+\eta_{1}}+e^{\xi_{1}+\eta_{2}}+e^{\xi_{2}+\eta_{1}}+e^{\xi_{2}+\eta_{2}}\right). \end{array}$$

Then combining (19) with (12a) yields

(20a) f(2)=k12(eξ1+η1+(π/2)i+θ13+eξ1+η2+(π/2)i+θ14) +k22(eξ2+η1+(π/2)i+θ23+eξ2+η2+(π/2)i+θ24),

(20b) s(2)=l12(eξ1+η1+(π/2)i+θ13+eξ2+η1+(π/2)i+θ23) +l22(eξ1+η2+(π/2)i+θ14+eξ2+η2+(π/2)i+θ24).

Substituting (18b) and (20a) into (10b), by some computations, we obtain

(21) g(3)=l12eξ1+ξ2+η1+(π/2)i+θ13+θ23+θ12 +l22eξ1+ξ2+η2+(π/2)i+θ14+θ24+θ12,

where

(22) eθ13=1(l1−k1)2,  eθ23=1(l1−k2)2,eθ14=1(l2−k1)2,  eθ24=1(l2−k2)2,eθ12=(k1−k2)2.

Then substituting (18b) and (20b) into (11b), by some computations, we obtain

(23) h(3)=k12eξ1+η1+η2+(π/2)i+θ13+θ14+θ34 +k22eξ2+η1+η2+(π/2)i+θ23+θ24+θ34,

where *e* ^*θ*_34_^ = (*l*_1_ − *l*_2_)^2^.  Substituting (21) and (23) into (12b) and (13b), we obtain

(24a) $$\begin{array}{c} f^{\left(4\right)}=\frac{k_{1}k_{2}}{4}e^{\xi_{1}+\xi_{2}+\eta_{1}+\eta_{2}+\pi i+\theta_{12}+\theta_{13}+\theta_{14}+\theta_{23}+\theta_{24}+\theta_{34}}, \end{array}$$

(24b) $$\begin{array}{c} s^{\left(4\right)}=\frac{l_{1}l_{2}}{4}e^{\xi_{1}+\xi_{2}+\eta_{1}+\eta_{2}+\pi i+\theta_{12}+\theta_{13}+\theta_{14}+\theta_{23}+\theta_{24}+\theta_{34}}. \end{array}$$

Assuming that *g* ^(*i*)^ = *h* ^(*i*)^ = *f* ^(*j*)^ = *s* ^(*j*)^ = 0, (*i* = 5,7,…, *j* = 6,8,…), one can find that (10a), (10b) and (10c)–(13a), (13b), and (13c) are still hold. Thus, let *ϵ* = 1, we have two-soliton solution for GDNLSE (2a) and (2b):

(25) q=(g(1)+g(3))(1+s(2)+s(4))(1+f(2)+f(4))2,r=(h(1)+h(3))(1+f(2)+f(4))(1+s(2)+s(4))2.

Figure 2 gives the interaction of two-soliton solution.

So, by the standard Hirota's approach, one can derive *N*-soliton (*N* = 1,2,…) in terms of *f*,  *g*,  *h*, and *s*:

(26a) $$\begin{array}{c} g_{N}\left(t,x\right)=\overset{}{\underset{\mu =0,1}{\sum }}A_{2}\left(\mu \right)\exp\left[\overset{2N}{\underset{j=1}{\sum }}\mu_{j}\xi_{j}^{\prime }+\overset{2N}{\underset{1\leq j<\rho }{\sum }}\mu_{j}\mu_{\rho }\theta_{j\rho }\right], \end{array}$$

(26b) $$\begin{array}{c} f_{N}\left(t,x\right)=\overset{}{\underset{\mu =0,1}{\sum }}A_{1}\left(\mu \right)\exp\left[\overset{2N}{\underset{j=1}{\sum }}\mu_{j}\xi_{j}^{\prime \prime }+\overset{2N}{\underset{1\leq j<\rho }{\sum }}\mu_{j}\mu_{\rho }\theta_{j\rho }\right], \end{array}$$

(26c) $$\begin{array}{c} h_{N}\left(t,x\right)=\underset{\mu =0,1}{\sum }A_{3}\left(\mu \right)\exp\left[\overset{2N}{\underset{j=1}{\sum }}\mu_{j}\eta_{j}^{\prime }+\overset{2N}{\underset{1\leq j<\rho }{\sum }}\mu_{j}\mu_{\rho }\theta_{j\rho }\right], \end{array}$$

(26d) $$\begin{array}{c} s_{N}\left(t,x\right)=\underset{\mu =0,1}{\sum }A_{1}\left(\mu \right)\exp\left[\overset{2N}{\underset{j=1}{\sum }}\mu_{j}\eta_{j}^{\prime \prime }+\overset{2N}{\underset{1\leq j<\rho }{\sum }}\mu_{j}\mu_{\rho }\theta_{j\rho }\right], \end{array}$$

where

(27a) $$\begin{array}{c} \xi_{j}=-k_{j}x+\omega_{j}t+\xi_{j}^{\left(0\right)},\;\omega_{j}=k_{j}^{2},\;\;\left(j=1,2,\ldots ,N\right) \end{array}$$

(27b) $$\begin{array}{c} \eta_{j}=l_{j}x+\sigma_{j}t+\eta_{j}^{\left(0\right)},\;\sigma_{j}=-l_{j}^{2}, \end{array}$$

(27c) ξj′=ξj,  ξN+j′=ηj+ln⁡⁡lj+π2i,ξj′′=ξj+ln⁡⁡kj+π2i,  ξN+j′′=ηj,

(27d) ηj′=ξj+ln⁡⁡kj+π2i,  ηN+j′=ηj,ηj′′=ηj+ln⁡⁡lj+π2i,  ηN+j′′=ξj,

(27e) $$\begin{array}{c} e^{\theta_{j,N+\rho }}=\frac{1}{{\left(k_{j}-l_{\rho }\right)}^{2}},\;\left(j,\rho =1,2,\ldots ,N\right), \end{array}$$

(27f) $$\begin{array}{c} e^{\theta_{j,\rho }}={\left(k_{j}-k_{\rho }\right)}^{2},\;\left(j<\rho =2,3,\ldots ,N\right), \end{array}$$

(27g) $$\begin{array}{c} e^{\theta_{N+j,N+\rho }}={\left(l_{j}-l_{\rho }\right)}^{2},\;\left(j<\rho =2,3,\ldots ,N\right). \end{array}$$

*k*_*j*_, *l*_*j*_, *ξ*_*j*_^(0)^, *η*_*j*_^(0)^ are all arbitrary constants; *A*_1_(*μ*), *A*_2_(*μ*), *A*_3_(*μ*) take over all possible combinations of *μ*_*j*_ = 0,1  (*j* = 1,2,…, 2*N*) and satisfy the following condition:

(28) ∑j=1Nμj=∑j=1NμN+j,  ∑j=1Nμj=∑j=1NμN+j+1,1+∑j=1Nμj=∑j=1NμN+j,

respectively.

We replace *e* ^*ξ*_1_^(0)^^, *e* ^*ξ*_2_^(0)^^, *e* ^*η*_1_^(0)^^ and *e* ^*η*_2_^(0)^^ by (*αe* ^*ξ*_1_^(0)^^/(*k*_1_ − *k*_2_)), (*αe* ^*ξ*_2_^(0)^^/(*k*_2_ − *k*_1_)), (*βe* ^*η*_1_^(0)^^/(*l*_1_ − *l*_2_)) and (*βe* ^*η*_2_^(0)^^/(*l*_2_ − *l*_1_)) (*α* and *β* are arbitrary real constants), respectively. Then the two-soliton solution (25) under the limit of *k*_2_ → *k*_1_, *l*_2_ → *l*_1_ leads to the limit solution

(29) $$\begin{array}{c} q=\frac{\overset{-}{g}\;\overset{-}{s}}{{\overset{-}{f}}^{2}},\;\;r=\frac{\overset{-}{h}\;\overset{-}{f}}{{\overset{-}{s}}^{2}}, \end{array}$$

where

(30a)g−=(2k1t−x)eξ1−l1α2e2ξ1+η1+(π/2)i(l1−k1)4,h−=(−2l1t+x)eη1−k1β2eξ1+2η1+(π/2)i(l1−k1)4,(30b)f−=1+2k1(l1−k1)2eξ1+η1+(π/2)i+α2β2k124(l1−k1)8e2ξ1+2η1+πi,(30c)s−=1+2l1(l1−k1)2eξ1+η1+(π/2)i+α2β2l124(l1−k1)8e2ξ1+2η1+πi.

*α* and *β* are arbitrary constants. This is the so-called one-double-pole solution. This kind of limit procedure can be found in [21, 22], which builds a bridge between Hirota's approach and the inverse scattering transform on the level of double-pole solution. Zhou and the coauthors find that the limit solutions for classical 2*N*-solitons are nothing but the *N*-double-pole solutions [23]. 

Now, we consider the derivative nonlinear Schrödinger equation (1). We shall give its bilinear equation and *N*-soliton solutions by reduction. Setting *r* = q* = u* and replacing *t* by −*it* and *x* by −*x* in (2a) and (2b), one can find that (2a) and (2b) reduce to DNLSE (1). Taking *s* = f*, *h* = g* and replacing *t* by −*it* and *x* by −*x*, (7a), (7b), (7c), and (7d) reduce to the bilinear forms of DNLSE (1):

(31a) $$\begin{array}{c} \left(iD_{t}+D_{x}^{2}\right)g\cdot f=0, \end{array}$$

(31b) $$\begin{array}{c} \left(iD_{t}+D_{x}^{2}\right)f\cdot f^{*}=0, \end{array}$$

(31c) $$\begin{array}{c} D_{x}f\cdot f^{*}=\frac{i}{2}gg^{*}, \end{array}$$

which can be also directly obtained from (1) through the transformation *u* = (gf*/*f* ^2^). If we take *l*_*j*_ = −*k*_j_*, *η*_*j*_^(0)^ = *ξ*_*j*_^(0)∗^ in (26a), (26b), (26c), and (26d) and (27a), (27b), (27c), (27d), (27e), (27f), and (27g), then *η*_*j*_ = *ξ*_j_*, *e* ^*θ*_*j*,*N*+*ρ*_∗^ = *e* ^*θ*_*ρ*,*N*+*j*_^, *e* ^*θ*_*j*,*ρ*_∗^ = *e* ^*θ*_*N*+*j*,*N*+*ρ*_^. Thus we can also have *s* = f*, *h* = g*, and obtain *N*-soliton solutions of DNLSE (1) by reduction:

(32a) $$\begin{array}{c} g_{N}\left(t,x\right)=\underset{\mu =0,1}{\sum }A_{2}\left(\mu \right)\exp\left[\overset{2N}{\underset{j=1}{\sum }}\mu_{j}\xi_{j}^{\prime }+\overset{2N}{\underset{1\leq j<\rho }{\sum }}\mu_{j}\mu_{\rho }\theta_{j\rho }\right], \end{array}$$

(32b) $$\begin{array}{c} f_{N}\left(t,x\right)=\underset{\mu =0,1}{\sum }A_{1}\left(\mu \right)\exp\left[\overset{2N}{\underset{j=1}{\sum }}\mu_{j}\xi_{j}^{\prime \prime }+\overset{2N}{\underset{1\leq j<\rho }{\sum }}\mu_{j}\mu_{\rho }\theta_{j\rho }\right], \end{array}$$

where

(33a)ξj=kjx−ikj2t+ξj(0),ξj′=ξj,ξN+j′=ξj∗+ln⁡⁡(−kj∗),(33b) ξj′′=ξj+ln⁡⁡kj, ξN+j′′=ξj∗, (j=1,2,…,N),(33c)eθj,N+ρ=12(kj+kρ∗)2, (j,ρ=1,2,…,N),(33d)eθj,ρ=2(kj−kρ)2, (j<ρ=2,3,…,N),

*k*_*j*_, *ξ*_*j*_^(0)^ are all arbitrary constants; *A*_1_(*μ*), *A*_2_(*μ*) take over all possible combinations of *μ*_*j*_ = 0,1  (*j* = 1,2,…, 2*N*) and satisfy the condition (28). If replacing *k*_*j*_ for *p*_*j*_, *e* ^*ξ*_*j*_^(0)^^ for *α*_*j*_, and *t* for −*t*, (32a) and (32b) are in accord with the *N*-soliton solutions in [14], where the solutions of DNLSE (1) are reduced by a multicomponent modified nonlinear Schrödinger equation. Dynamics for one- and two-soliton solutions for DNLSE (1) are described in Figures 3 and 4.  Figure 4 depicts 2D plot of two-soliton of DNLSE.

In summary, we present multisoliton solutions for a general nonlinear Schrödinger equation with derivative by Hirota's approach. By reductions, we also directly obtain the multisoliton solutions for nonlinear derivative Schrödinger equation. We demonstrate that the solitons of general nonlinear Schrödinger equation with derivative and nonlinear derivative Schrödinger equations result in elastic scattering.

## Figures and Tables

**Figure 1:**
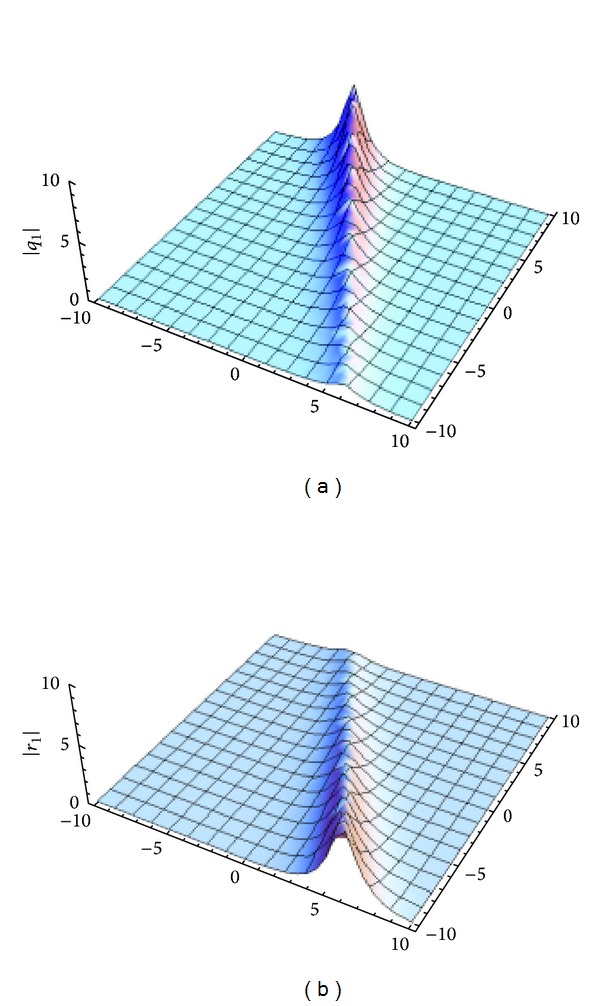
The shape of the one-soliton solution of GDNLSE given by (17). *q*_1_ and *r*_1_ with *k*_1_ = 1 + 0.3*i*, *l*_1_ = −1 + 0.2*i*, and *ξ*_1_^(0)^ = *η*_1_^(0)^ = 0. (a) |*q*_1_|, (b) |*r*_1_|.

**Figure 2:**
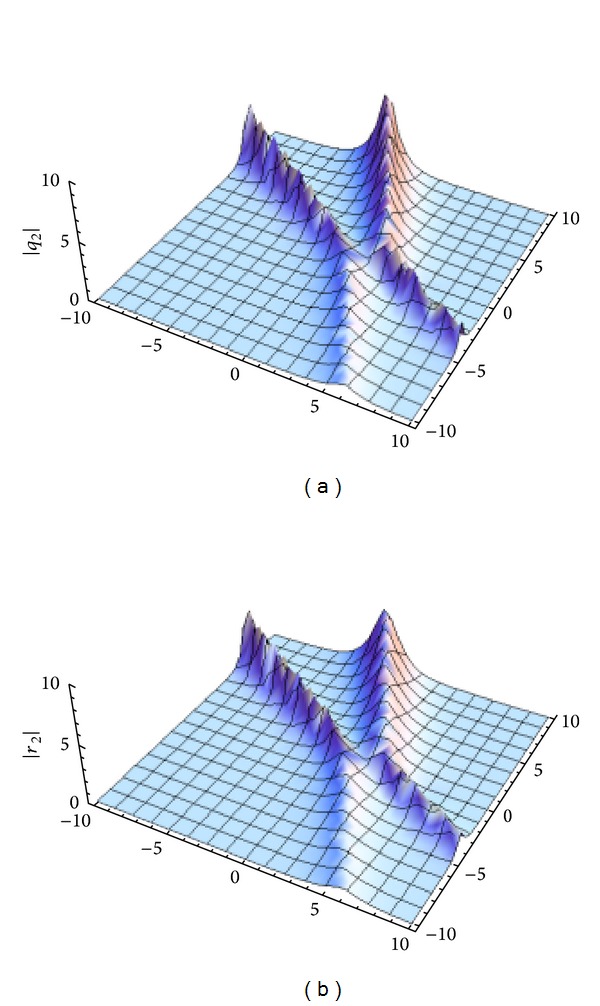
The interaction of two-soliton solution of GDNLSE given by (6). *q*_2_ and *r*_2_ with *k*_1_ = 1 + 0.3*i*,  *k*_2_ = 1 + 0.9*i*, *l*_1_ = −1 + 0.2*i*,  *l*_2_ = −1 + 0.8*i*, and *ξ*_1_^(0)^ = *ξ*_2_^(0)^ = *η*_1_^(0)^ = *η*_2_^(0)^ = 0. (a) |*q*_2_|, (b) |*r*_2_|.

**Figure 3:**
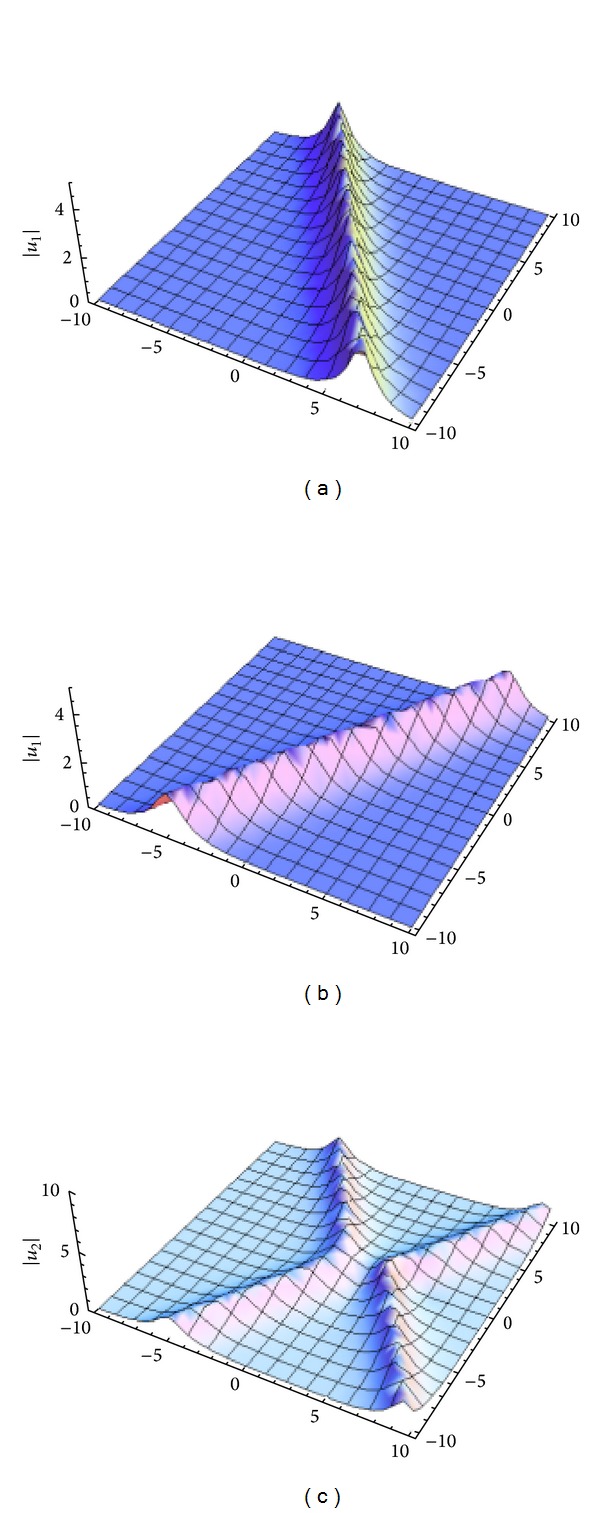
The shape of the one-soliton and the interaction of two-soliton of DNLSE (1). (a) One-soliton *u*_1_ with *k*_1_ = 1 + 0.3*i* and *ξ*_1_^(0)^ = 0, (b) one-soliton *u*_1_ with *k*_1_ = 1 − 0.3*i* and *ξ*_1_^(0)^ = 0, and (c) two-soliton *u*_2_ with *k*_1_ = 1 + 0.3*i*, *k*_2_ = 1 − 0.3*i*, and *ξ*_1_^(0)^ = *ξ*_2_^(0)^ = 0.

**Figure 4:**
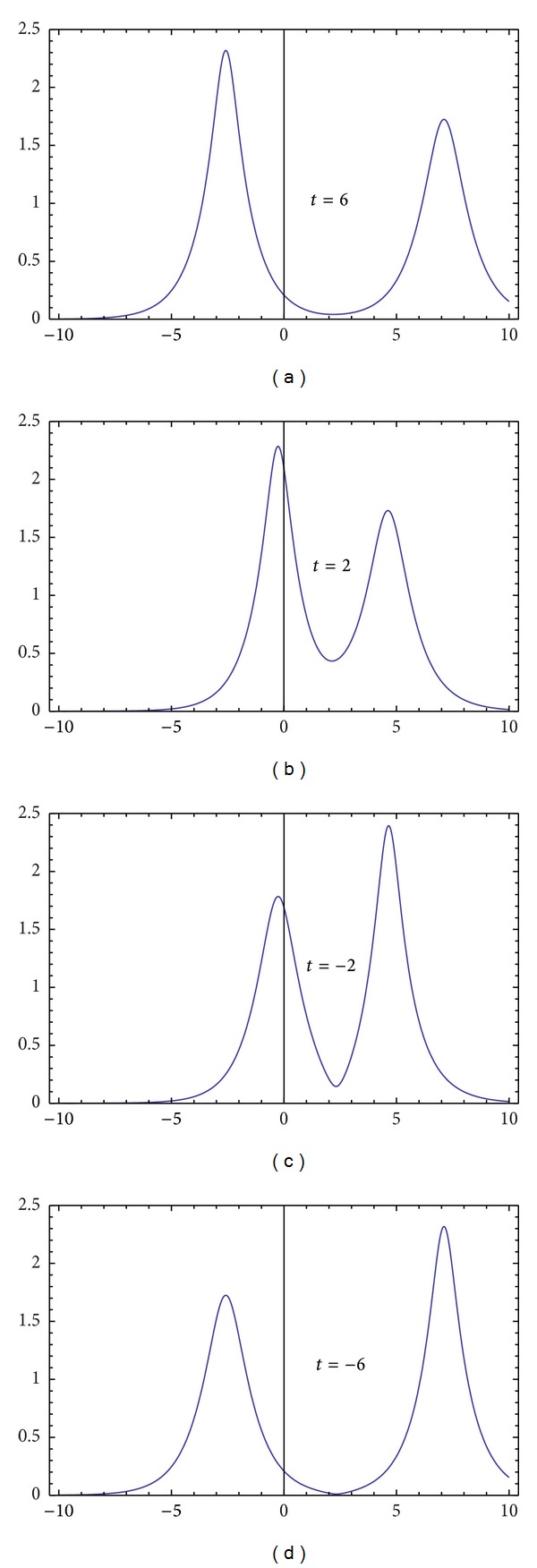
The 2D plot of two-soliton of DNLSE. *q*_2_ with *k*_1_ = 1 + 0.3*i*, *k*_2_ = 1 − 0.3*i*, and *ξ*_1_^(0)^ = *ξ*_2_^(0)^ = 0 at *t* = 6, *t* = 2, *t* = −2, and *t* = −6.
